# Bickerstaff Brainstem Encephalitis Presenting With Negative Anti-GM1 and Anti-GQ1B Antibodies

**DOI:** 10.7759/cureus.61653

**Published:** 2024-06-04

**Authors:** Alice Warcup, Guilherme Movio, Saikat Dhar, Christopher Till

**Affiliations:** 1 Medical Education, Lancaster Medical School, Lancaster, GBR; 2 Neurology, Royal Preston Hospital, Lancashire Teaching Hospitals NHS Foundation Trust, Lancashire, GBR; 3 Intensive Care, Blackpool Victoria Hospital, Blackpool, GBR

**Keywords:** neuro-critical care, invasive mechanical ventilation, neurology and critical care, intensive care medicine, autoimmune neuromuscular disease

## Abstract

A woman in her 60s initially presented with rapid-onset left-sided hemiparesis with later development of slurred speech and left-sided facial droop. Despite ruling out common causes, her condition rapidly progressed with the development of bilateral proximal weakness, ophthalmoplegia, ataxia, and pyramidal signs eventually leading to a cardiorespiratory arrest. Extensive investigations, including computerised tomography (CT), magnetic resonance imaging (MRI), and lumbar puncture (LP), were negative for infectious or vascular aetiologies. Nerve conduction studies (NCS) revealed severe peripheral nerve damage, and despite a provisional diagnosis of Guillain-Barré Syndrome (GBS), the clinical picture aligned more with Bickerstaff Brainstem Encephalitis (BBE) given the central nervous system (CNS) involvement, despite negative anti-GM1 and anti-GQ1b autoantibodies. Treatment involved ventilatory support, immunoglobulins, and steroids. This case report describes a rare and challenging presentation of BBE and reminds clinicians to have a systematic approach to a patient presenting with rapid onset neurological symptoms and that BBE is a clinical diagnosis.

## Introduction

Bickerstaff Brainstem Encephalitis (BBE) is a rare neurological disorder characterised by a triad of symptoms, namely ophthalmoplegia, ataxia, and impaired consciousness or pyramidal signs [1]. First described by Bickerstaff and Cloake in 1951, its prevalence is notably low, with only a limited number of cases reported worldwide [2]. BBE is thought to be on a spectrum with other neurological disorders such as Guillain-Barré Syndrome (GBS) and Miller-Fisher Syndrome (MFS), which makes its identification difficult [3]. BBE, GBS, and MFS are thought to be post-infective and although the exact aetiology remains unclear, BBE is thought to be due to autoimmune mechanisms, in particular molecular mimicry [4]. We present a case report in which rapidly progressive unilateral and then later bilateral weakness presented alongside ophthalmoplegia and ataxia, where a diagnosis of BBE was made. This case report aims to contribute to the scarce literature on BBE, including its clinical features, diagnostic challenges, and therapeutic considerations.

## Case presentation

A woman in her 60s, with no significant medical, social, or family history, presented to the Emergency Department (ED) after a fall whilst attempting to mobilise from her bed. She reported flu-like symptoms two weeks prior to this. She described a sudden onset of left-sided arm and leg weakness, difficulty coordinating her walking and writing, and a sensation of tongue swelling. No loss of consciousness occurred although the patient was drowsy. The patient also complained of a moderate, bilateral persistent headache coinciding with the onset of weakness. There was no history of neck pain or hyperextension injury. On arrival at the ED, her vital signs were within normal ranges. However, given the reported unilateral weakness and slurred speech, a comprehensive stroke assessment was conducted.

The neurological examination using the muscle power scale (MRC) showed bilateral lower limb weakness (4/5 MRC), upper limb weakness (3/5 MRC), preserved head and neck movement, eye-opening, full visual fields, and eye movements. No nystagmus was observed, and the patient felt drowsy. Tone, sensation, and reflexes were intact at this stage. However, mobilisation required assistance from two individuals due to ataxia. An urgent CT of the head yielded no acute findings. CTA was also normal. Considering the diagnostic uncertainty, the decision was made to admit the patient for further investigation.

Approximately 8 hours later, whilst awaiting a ward bed, the patient deteriorated further with a left-sided facial droop and difficulty swallowing. In addition, she experienced heaviness in the tongue and drooling. The weakness had progressed at this stage from unilateral to bilateral lower limb weakness. The proximal lower limb weakness was recorded as 3/5 MRC and the feet maintained power at 5/5 MRC bilaterally. There was no documented sensory level, and complete loss of bilateral arm power (0/5 MRC), along with impaired head and neck movement and difficulty opening the eyes. Ophthalmic examination revealed reactive pupils but no accommodation. Bilateral adduction deficits were present without abduction nystagmus. Although the patient did not report diplopia, wall-eyed bilateral intra-nuclear ophthalmoplegia was noted. The patient also had upgoing plantars bilaterally. At this stage, hypotonia was noted, but dorsal column and spinothalamic sensation remained intact and there was eventual loss of deep tendon reflexes. There was no mention of either Hoffman’s sign or the presence of clonus. A repeat CT was performed to rule out a new acute intracranial event; however, it demonstrated no change from the initial scan. The patient was moved to an acute stroke ward.

Subsequently, 12 hours following this, the patient experienced an unresponsive episode, leading to respiratory arrest and progression to a pulseless electrical activity (PEA) cardiac arrest. Prompt cardiopulmonary resuscitation (CPR) was initiated, requiring two cycles of CPR before achieving return of spontaneous circulation (ROSC). It was thought that the patient developed a decreased consciousness level which led to a loss of airway tone, and subsequently culminating in the cardiorespiratory arrest secondary to hypoxia. Given the clinical presentation, a decision was made to proceed with a rapid sequence induction, and the patient was transferred to the local intensive care unit (ICU).

Investigations

Upon initial assessment in the ED, the patient underwent standard blood investigations as shown in Table 1.

**Table 1:** Standard blood investigations undertaken in the Emergency Department at admission HDL: high density lipoprotein; eGFR: estimated glomerular filtration rate; HbA1c: haemoglobin A1C; L: liter; g/L: grams per liter; fL: femtoliter; pg/cell: picogram per cell; mmol/L: millimole per liter; mU/L^2^: milliunits per liter squared; mmol/mol: millimole per mole; U/L: units per liter

Parameter	Value	Reference Range and units
White blood count	5.9	3.6-11.0 x10^9^/L
Red blood count	4.57	3.8-5.8 x10^12^/L
Haemoglobin	111	115-165 g/L
Packed cell volume	0.367	0.360-0.440 L/L
Mean corpuscular volume	80.4	80-100 fL
Mean corpuscular haemoglobin	24.3	27-32 pg/cell
Mean corpuscular haemoglobin concentration	302	320-365 g/L
Red cell distribution width	17.2	11.5-15.0 %
Platelets	240	140-400 x10^9^/L
Neutrophils	3.81	1.8-7.5 x10^9^/L
Lymphocytes	1.5	1.0-4.0 x10^9^/L
Monocytes	0.25	0.2-0.8 x10^9^/L
Eosinophils	0.26	0.1-0.4 x10^9^/L
Basophils	0.01	0.02-0.1 x10^9^/L
Non-HDL cholesterol	4.05	<3 mmol/L
HDL cholesterol	1.55	>1 mmol/L
Total cholesterol/HDL ratio	3.6	<4
Triglyceride	3.9	0.55-1.90 mmol/L
eGFR	67	>90
Total iron binding capacity	82.1	45-81 mmol/L
Iron	28.1	4.6-30.4 mmol/L
Thyroid stimulating hormone	2.16	0.4-4.0 mU/L^2^
HbA1c	35	<42 mmol/mol
Sodium	140	133-146 mmol/L
Potassium	4.6	3.5-5.5 mmol/L
Urea	4.5	2.5-7.8 mmol/L
Creatinine	82	45-84 mmol/L
Total protein	69	60-78 g/L
Albumin	42	35-50 g/L
Bilirubin	12	<21 mmol/L
Alkaline phosphatase	94	30-130 U/L
Adjusted calcium	2.36	2.2-2.6 mmol/L
Alanine transaminase	14.7	<33 U/L

**Figure 1:**
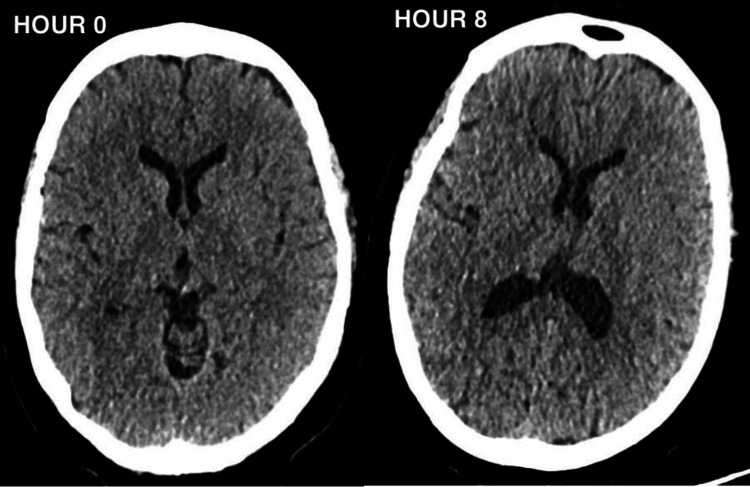
Showing the CT head at presentation (Hour 0) and eight hours later (Hour 8).

A 12-lead electrocardiogram (ECG) revealed no signs of acute cardiac pathology. CT head scans performed upon admission and approximately 8 hours later to exclude acute cerebral events showed no intra or extra-axial haemorrhage, infarction, or space-occupying lesions (Figure 1). The patient also underwent a follow-up MRI head scan which exhibited no abnormal enhancement of the brain or spinal cord. After admission to the ICU, a lumbar puncture (LP) was performed, revealing results shown in Table 2.

**Table 2:** Lumbar puncture results undertaken during the ICU admission cells/mL: cells per milliliter; g/L: grams per liter; mmol/L: millimole per liter

Parameter	Value	Reference Range and units
White cell count	4.00	<5 cells/mL
Red cell count	2.00	<1 cells/mL
Gram stain	No organisms grown	-
Protein	0.48	<0.45 g/L
Glucose	3	2.8-4.4 mmol/L

A wide range of microbiological tests were performed to rule out infective causes of the patient’s symptoms. These included: Norovirus, Rotavirus, Campylobacter, Salmonella, Shigella, *Escherichia coli*, *Clostridium difficile*, Giardia, Vibrio cholera, Cryptosporidium, Human Immunodeficiency Virus, Epstein-Barr virus, and a viral hepatitis screen. All of the above were negative.

Considering the possibility of GBS based on discussions with the local neurology centre, an autoantibody test was conducted four days after admission to the ICU, results are shown in Table 3. These results being negative make Anti-myelin oligodendrocyte glycoprotein (MOG) syndromes and neuromyelitis optica spectrum disorders unlikely [5].

**Table 3:** Specialist neurological autoantibody blood test IgG: immunoglobulin G; IgM: immunoglobulin M; GM1: ganglioside monosialic; GD1a: ganglioside D1a; Anti-MOG: anti-myelin oligodendrocyte glycoprotein; GQ1b: Ganglioside Q1b

Parameter	Value
GM1 IgG	negative
GM1 IgM	negative
GD1a IgG	negative
GD1a IgM	negative
Anti- GQ1b	negative
Anti- Mog autoantibodies	negative
Anti-Aquaporin autoantibodies	negative

**Figure 2:**
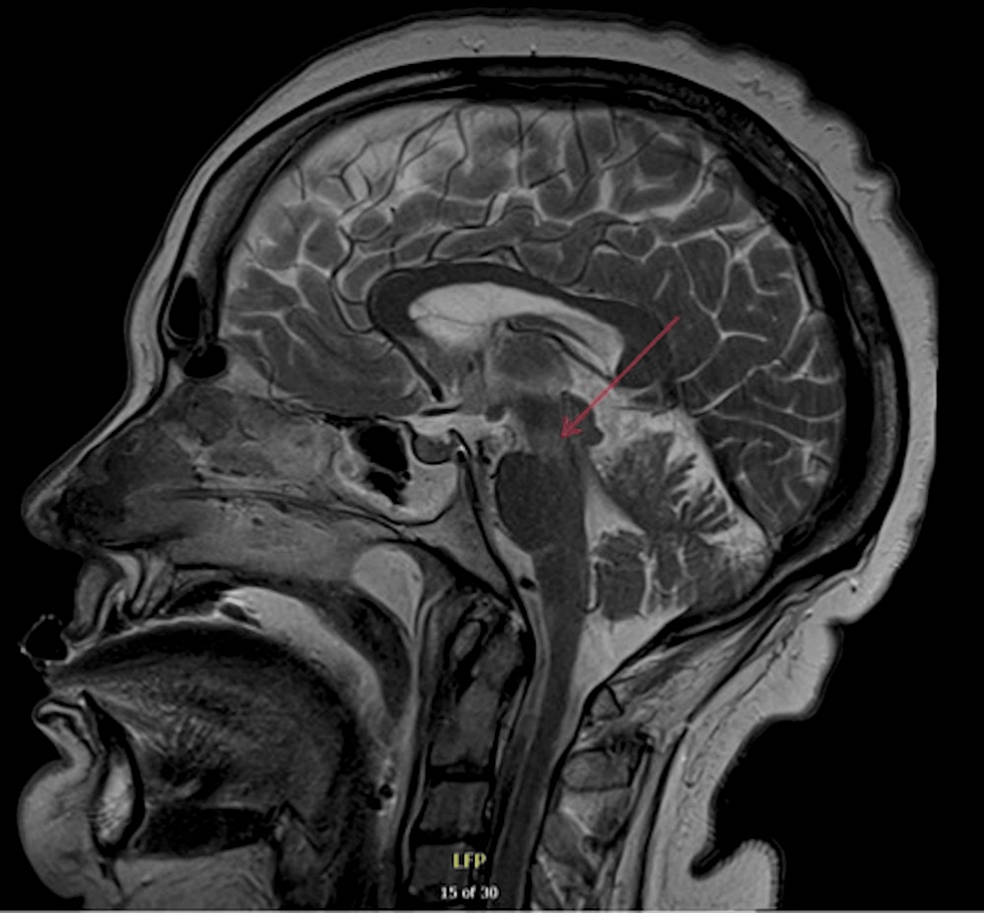
MRI head showing T2 hyperdensity at the mesocepahalopontine junction (red arrow) T2: T2 weighted image

The decision was made to perform a percutaneous tracheostomy, and then transfer the patient to a local tertiary neurological centre for further neurological evaluation. An electroencephalogram (EEG) was performed which revealed no evidence of non-convulsive status epilepticus but indicated cortical dysfunction consistent with moderate encephalopathy. The EEG showed no periodic lateralised epileptiform discharges or generalised periodic epileptiform discharges. Nerve conduction studies (NCS) and electromyography (EMG) were also conducted and the report detailed in Tables 4-6, indicated severe peripheral nerve damage in the cranial segment and upper limbs. No spontaneous activity was observed in any upper limb muscles. Motor conduction in the lower limbs was described as normal, but there was evidence of sensory deafferentation. To rule out spinal cord pathology an MRI spine was conducted which showed no spinal cord injury. While at the tertiary neurological centre, an MRI brain showed T2 hyperintensity at the mesocepahalopontine junction, shown in Figure 2. The MRI showed no contrast enhancement as well as no evidence to suggest brainstem stroke. Given the clinical neurology examination, bulbar symptoms, and MRI results, the diagnosis was narrowed to BBE. 

**Table 4:** Sensory nerve conduction results

Nerve/sites	Response
Left median and ulnar nerve/ digit 2,3,5	No response
Left radial/anatomical snuffbox	No response
Left lateral antebrachial cutaneous/elbow	No response
Left sural/lower leg	No response
Right sural/lower leg	No response

**Table 5:** Motor nerve conduction results ms: millisecond; P-P amplitude: Peak-to-peak amplitude; mV: millivolts; mm: millimeters; m/s: meters per second

Nerve/Sites	Onset (ms)	P-P Amplitude (mV)	Segments	Latency Difference (ms)		Distance (mm)		Velocity (m/s)
Left Median – Recording Abductor pollicis brevis, Stimulation Wrist, Elbow, Axilla								
Wrist	3.3	0.1	Wrist – Elbow	-4.7	240		51.0	
Elbow	8.0	0.1	Elbow – Axilla	-	-		-	
Axilla	-	-	Axilla – Erbs Point	-	-		-	
Left Ulnar – Recording Abductor digiti minimi, Stimulation Wrist, Elbow, Axilla								
Wrist	5.6	0.0	Wrist – Below Elbow	-	-		-	
Right Peroneal – Recording Extensor digitorum brevis, Stimulation Ankle, Fibula, Popliteal Fossa								
Ankle	4.5	6.4	Ankle – Neck of Fibula	-7.1	335		46.9	
Neck of Fibula	11.6	5.7	Neck of Fibula – Popliteal Fossa	-	-		-	
Right Tibial – Recording Abductor hallucis, Stimulation Ankle, Knee								
Ankle	4.3	8.7	Ankle – Knee	-7.3	370		50.6	
Left Peroneal – Recording Extensor Digitorum brevis, Stimulation Ankle, Fibula, Popliteal Fossa								
Ankle	4.8	9.6	Ankle – Neck of Fibula	-	-		-	
Neck of Fibula	12.1	6.7	Neck of Fibula –Popliteal Fossa	-	-		-	
Right Tibial – Recording Abductor hallucis, Stimulation Ankle, Knee								
Ankle	3.9	10.2	Ankle – Knee	-	-		-	
Left Facial – Nasalis, Orbicularis Oculi, Orbicularis Oris								
Postauricular	No Response	No Response	-	-	-		-	
Left Facial – Nasalis, Orbicularis Oculi, Orbicularis Oris								
Postauricular	No Response	No Response	-	-	-		-	

**Table 6:** Electromyography results MUAPs: motor unit action potentials

Insertional Activity		Spontaneous Activity			Volitional MUAPs				Maximum volitional Activity	
Muscle	Insertional	Fibrillation	+ Wave	Fasciculation	Duration	Amplitude	Polyphasic potential	Recruitment	Pattern	Effort
Right Tibialis anterior	Normal	None	None	None	Normal	Normal	None	Normal	Moderate	Max
Left Tibialis anterior	Normal	None	None	None	Normal	Normal	None	Normal	Moderate/Discrete	Max
Left First dorsal interosseous	Normal	None	None	None	-	-	-	-	None	Max
Left Abductor pollicis brevis	Normal	None	None	None	-	-	-	-	None	Max
Left Extensor digitorum communis	Normal	None	None	None	-	-	-	-	None	Max
Left Deltoid	Normal	None	None	None	-	-	-	-	None	Max
Left frontalis	Increased	1+	1+	None	Normal	Normal	None	Reduced	Discrete	Max
Left Orbicularis oris	Increased	None	None	None	-	-	-	-	None	Max
Right Frontalis	Normal	None	None	None	Normal	Normal	Few	Reduced	Discrete	Max
Left Masseter	Increased	None	1+	None	-	-	-	-	None	Max
Right Masseter	Normal	None	None	None	Normal	Normal	None	Normal	Full	Max
Right Mentalis	Normal	None	None	None	-	-	-	-	None	Max
Right Orbicularis oculi	Increased	None	1+	None	-	-	-	-	None	Max
Left Orbicularis oculi	Increased	None	None	None	Normal	Normal	None	Normal	Full	Max

Treatment

Ventilatory support played a central role. The patient was initially intubated and mechanically ventilated for airway protection. Once differentials such as an acute cerebrovascular event, and central nervous system (CNS) infections were ruled out, and the provisional diagnosis of GBS was made, treatments became more targeted. The patient received five doses of 0.4 g/kg of intravenous immunoglobulin (IVIG), combined with once-daily 1-gram intravenous methylprednisolone. However, despite receiving the recommended five days of this treatment combination, the patient exhibited little to no improvement in symptoms. It is crucial to note that the utilisation of IVIG and steroids in BBE is based on a limited evidence base, associated with the more common variant, GBS. 

A percutaneous tracheostomy was performed shortly after admission to the local ICU, given the suspicion of necessitating a long-term respiratory wean due to GBS which causes profound weakness of respiratory muscles and multiple failed extubation attempts [6].

Once the diagnosis was narrowed to BBE, the team felt that there were no further pharmacological interventions that could be given to treat BBE. The focus of treatment therefore became supportive of the patient’s neurological status. The patient was then placed on a comprehensive physiotherapy plan aimed at gradual weaning from ventilator support. The patient received pharmacological treatments for excessive airway secretions such as glycopyrronium bromide, a muscarinic antagonist, to reduce the volume of airway secretions. To manage salivary gland secretions, onabotulinum toxin A injections were trialled in the submandibular and parotid glands. This approach aimed to mitigate excessive salivation associated with the patient's condition and support ventilatory weaning.

The multifaceted approach to treatment, incorporating ventilatory support, pharmacological interventions, and physiotherapy, reflects the comprehensive and individualised management strategy tailored to address the specific challenges posed by BBE in this patient.

Outcome and follow-up

The patient continues to receive supportive management for BBE. The observed weakness persists in the head, neck, and upper limbs, with partial improvement noted in lower limb mobility. The ongoing process of long-term tracheostomy weaning, and physiotherapy remains integral to the patient's future care plan. 

The extent of recovery for this patient is challenging to predict due to the limited available reports on BBE, adding an element of uncertainty to the prognosis. The patient is anticipated to face an extended period of recovery, emphasising the importance of a comprehensive and individualised approach to their ongoing care.

## Discussion

This case accentuates the diagnostic challenges linked to BBE and highlights the significance of considering a diverse range of differentials for accurate and timely management. The case initially featured a unilateral weakness which rapidly developed over hours into a distinctive pattern of bilateral weakness affecting muscles above the knees, upper limbs, neck, and face.

An acute cerebrovascular event emerged as an initial consideration, as it can manifest with rapid-onset weakness. However, the bilateral progression of weakness observed in this patient differed from the typical unilateral involvement associated with stroke [7]. Despite this, it needed to be ruled out at first instance. The CT and MRI scans of the head revealed no evidence of haemorrhage or infarct, diminishing the likelihood of an acute cerebrovascular event. These also ruled out neoplastic space-occupying lesions.

Neurological infections, particularly viral and bacterial encephalitis, were explored as another potential cause of rapidly progressive weakness. Despite the absence of fever and intact cognitive function, CSF analysis showed no indications of infection, ruling out encephalitis as a primary diagnosis [8]. Botulism, characterised by descending weakness of head and neck muscles, was considered as well. However, the ascending pattern of weakness observed in this patient contradicted the expected presentation of botulism [9]. Incremental NCS further substantiated this, by confirming no electrophysiologic evidence for botulism. Lyme disease, known to induce weakness in facial muscles, was also contemplated [10]. Nevertheless, the absence of risk factors such as exposure to tick bites, skin rashes, or fever, coupled with negative serologic testing for Borrelia burgdorferi, lessened the likelihood of Lyme disease in this patient.

Once acute cerebrovascular events and neurological infections were excluded, GBS became the focus of investigations. On examination, the patient presented with progressive bilateral proximal weakness affecting the lower limbs, progressing to the upper limbs and then facial muscles. The patient also reported previous flu-like symptoms two weeks prior to admission, which the team suspected could have been a trigger to an autoimmune process. The CSF revealed a protein level of 0.48 g/L which corroborates this. Therefore, a provisional diagnosis of GBS was made. Once EMG and NCS studies were conducted, the consultant neurophysiologist interpreted the results (Tables 1-3) as showing an overall pattern consistent with MFS, however advised to correlate with clinical findings for potential brainstem encephalitis. In addition, MRI findings shown in Figure 2 correlate with BBE due to the hyperintensity seen in the brainstem. Although the GM1, GD1a, and GQb1 autoantibodies were negative, it is reported that only 66% of patients with BBE are anti-GQ1b antibody positive, and only 10% and 13% are associated with GM1 and anti-GD1a antibodies, respectively [11]. The clinical picture of ataxia, ophthalmoplegia and loss of consciousness, alongside clear CNS involvement demonstrated by the clinical examination, EEG and MRI allowed for a diagnosis of BBE. 

BBE, a rare autoimmune neurological condition impacting both the peripheral and central nervous systems, is viewed as a variant within the spectrum of immune-mediated polyneuropathy conditions, including GBS and MFS. Characterised by distinctive clinical features such as bilateral weakness in muscles of the lower limbs, upper limbs, neck, and face. BBE presents a triad of recognized symptoms: ophthalmoplegia, ataxia, and consciousness disturbance, along with pyramidal signs. Notably, BBE involves the CNS, unlike MFS and GBS, which typically spare CNS involvement.

Situated on a spectrum with GBS and MFS, BBE poses challenges in differentiation and subsequent diagnosis. The pathological mechanism of molecular mimicry is a common thread among these conditions, where an immune response triggered by infection may cross-react with host nerves, leading to demyelination and axonal damage and manifesting as characteristic motor and sensory deficits [2]. Additionally, all three conditions are associated with post-infectious factors; in this instance, the patient's upper respiratory tract infection serves as a presumed trigger [12].

This case aligns most closely with BBE, diverging from MFS and GBS due to evident CNS involvement and the presence of pyramidal signs. EEG results revealed cortical dysfunction consistent with moderate encephalopathy, MRI showed brainstem involvement and these investigations coupled with an upgoing plantar sign, make BBE more likely. Whilst the CNS involvement in BBE remains poorly understood, it is theorised to be linked to the disruption of the blood-brain barrier [13].

Guidelines for treating BBE are relatively scarce. The predominant approach aligns with that of GBS, involving either IVIG, as administered in this case, or plasmapheresis. Additionally, the patient received high-dose intravenous steroids, based on the limited evidence suggesting steroid treatment can enhance recovery [14]. However, a Cochrane review refrains from providing specific recommendations for the treatment of both BBE and MFS due to a scarcity of trials evaluating treatment efficacy in clinical settings [4]. 

BBE stands out as an exceptionally rare condition in the literature. Previous case reports highlight similar presentations characterised by a positive Babinski sign, ophthalmoplegia, and slurred speech [15]. Other documented cases provide valuable insights into the varied manifestations of BBE, showcasing instances of overlap with GBS, posterior reversible encephalopathy syndrome, and occurrences linked to chlamydia infection [16,17]. The pathophysiology of BBE is emphasised as autoimmune-mediated peripheral nerve damage triggered by antecedent infections, in various case reports [18]. The role of specific autoantibody testing, encompassing GM1, GD1a, and GQb1, is also discussed, despite the negative results observed in our patient [19]. The absence of specific autoantibodies in this case does not diminish the validity of the BBE diagnosis. 

Although our patient did not have any signs of infection on presentation, the literature reminds clinicians to consider early antibiotic intervention, recognising the potential co-existence of BBE with an active infection, such as *Salmonella *Dublin [20]. Antibiotics assume a critical role not only in managing sepsis but also in averting the onset of serious encephalopathy if there is suspicion of co-existing infection [20].

## Conclusions

In conclusion, the presented case shows the importance of thorough clinical assessment and systematic investigation in the evaluation of patients with rapid-onset neurological symptoms. While cerebrovascular events are often the primary concern, it is imperative for clinicians to maintain a broad differential diagnosis to encompass rare conditions like BBE, especially when the clinical picture does not align with more common presentations. This case highlights the distinction between BBE, MFS, and GBS. While sharing some clinical features and pathophysiological mechanisms, BBE stands out for the involvement of the CNS, presenting a triad of ophthalmoplegia, ataxia, and consciousness disturbance, alongside pyramidal signs. The absence of specific autoantibodies, such as anti-GM1 and anti-GQ1b, should not deter clinicians from considering a diagnosis of BBE, as their absence does not rule out the condition. Instead, correlation with the clinical presentation, neurological findings, and ancillary tests such as nerve conduction studies and electromyography are vital.
